# EphB2 represents an independent prognostic marker in patients with gastric cancer and promotes tumour cell aggressiveness

**DOI:** 10.7150/jca.38098

**Published:** 2020-02-21

**Authors:** Jie Yin, Zhilei Li, Lin Ye, Emily Birkin, Liting Li, Rui Xu, Guangyong Chen, Jiafu Ji, Zhongtao Zhang, Wen G. Jiang, Yuxin Cui

**Affiliations:** 1 Department of General Surgery, Beijing Friendship Hospital, Capital Medical University; Beijing Key Laboratory of Cancer Invasion and Metastasis Research and National Clinical Research Center for Digestive Diseases, 95 Yong-an Road, Xi-Cheng District, Beijing 100050, P.R. China; 2 Cardiff China Medical Research Collaborative, Division of Cancer and Genetics, Cardiff University School of Medicine, Heath Park, Cardiff CF14 4XN, UK; 3 Department of Pharmacy, Zhujiang Hospital of Southern Medical University, Guangzhou, 510282, P.R. China; 4 Key Laboratory of Carcinogenesis and Translational Research (Chinese Ministry of Education), Department of GI Surgery, Peking University Cancer Hospital and Institute, Beijing 100142, P.R. China; 5 Department of Pathology, Beijing Friendship Hospital, Capital Medical University;95 Yong-an Road, Xi-Cheng District, Beijing 100050, P.R. China

**Keywords:** EphB2, gastric cancer, survival, aggressiveness

## Abstract

Dysregulated expression of ephrin type-B receptor 2 (EphB2) has been linked with the development and progression of solid tumours. In the current study, we attempted to investigate the clinical relevance in GC and the effect of EphB2 expression on gastric cancer (GC) cells. EphB2 protein levels in GC and benign gastric tissues were determined using immunohistochemistry. EphB2 transcript expression in a GC cohort with GC tissue samples (n=171) and paired adjacent normal gastric tissues (n=97) was determined using qPCR. The EphB2 expression was over-activated using a CRISPR activator for the investigation of its cellular function. The expression levels of the EphB2 protein in the tumour tissues of tissue arrays were higher than the benign non-cancerous gastric tissues (P<0.05). EphB2 mRNA expression in GC tissues was also significantly elevated when compared with adjacent non-cancerous tissues (P<0.01). EphB2 activation promoted the migration and invasion abilities of the GC cell lines (P<0.01, respectively). In contrast, EphB2 activation significantly decreased the adhesion in GC cells (P<0.0001, respectively). The enrichment analysis of the correlated genes in a GC cohort indicates that EphB2 may function through mediating the cytokine-cytokine interaction, JAK-STAT and TP53 signaling pathways. In conclusion, EphB2 represents as a novel independent prognostic marker in GC. And activation of the EphB2 gene expression elevated the levels of migration and invasion, but suppressed adhesion of GC cells, indicating that EphB2 may act as a tumour promotor in GC. Our findings thus provide fundamental evidence for the consideration of the therapeutic potential of targeting EphB2 in GC.

## Introduction

The human ephrin receptors (Ephs) consist of 16 members and are categorised into two groups: Eph A (EphA1-8, 10) and EphB (EphB1-4, 6). The Eph receptors and their membrane-anchored ephrin ligands regulate tumourigenesis and tumour-associated angiogenesis 1, 2. The formation of an ephrin-Eph heterotetramer complex can trigger multiple downstream pathways, such as the Ras (R-Ras, H-Ras, N-Ras) and Rho (Rho, Rac1, Cdc42) family of small GTPases. Ephs play diverse and essential roles in cancer cells including modulating migration, proliferation, cytoskeletal dynamics, adhesion and invasion 3. Aberrant expression or mutations of Eph receptors are associated with tumour promotion or progression in lung, breast and colon cancer 4-6.

Among the five human EphB receptors, EphB2 has been extensively studied in several types of solid tumours. In glioma tumour, EphB2 regulates cell migration, growth and adhesion probably through the activation of the R-Ras pathway 7, 8. A higher level of EphB2 protein expression is associated with better clinical outcomes including both overall and recurrence-free survival of patients with colorectal cancer. However other studies suggest that EphB2 may be an invasion-driver gene in colorectal cancer 9, 10. Loss-of-function mutation of EPHB2 may also be involved in the progression and metastasis of prostate cancer 11.

Gastric cancer (GC) is the fourth most common cancer in men and the seventh most common cancer in women worldwide. Approximately 1 million new cases are diagnosed annually 12. GC is also the second most common cause of cancer-related deaths in the world. GC is often diagnosed at an advanced stage, and its prognosis is often poor with a median OS of less than 1 year. Therefore there is an unmet need for an enhanced understanding of the biology of this disease before developing more efficient therapies. The clinical significance EphB2 in GC remains controversial so far. It is reported that EphB2 is elevated in early-stage GC, but the loss of EphB2 expression tends to correlate with poor survival of the patients with GC 13, 14.

A previous study using tissue array also indicates that there may be a high level of EphB2 protein expression in intestinal adenocarcinoma of the stomach although there is no gene expression data presented to support it 15. There is also a report suggesting that high EPHB2 mutation rate may be associated with microsatellite instability in GC compared with endometrial tumours using a limited sample size 16. Therefore in this study, we aimed to evaluate the prognostic value and determine the functions of EphB2 in GC. A gastric tissue microarray was used to investigate the association of the EphB2 protein expression with gastric cancer by immunohistochemistry. We examined the gene expression of EphB2 in a GC cohort. The functional role of EphB2 was determined using gastric cancer cells following CRISPR-mediated EphB2 gene expression activation. The regulation pathways that EphB2 is involved were explored by bioinformatic analysis after pooling the significantly correlated genes with EphB2 in our GC cohort.

## Materials and methods

### Cell lines and culture conditions

Human GC cell lines AGS and HGC27 were purchased from the European Collection of Cell Cultures (ECACC; Salisbury, UK) and incubated at 37˚C, with 5% CO2 and 95% humidity. These cell lines were maintained in Dulbecco's modified Eagle's medium (DMEM) supplemented with 10% fetal calf serum (PAA Laboratories Ltd., Somerset, UK), and 1% penicillin, streptomycin and amphotericin B (Sigma-Aldrich, Dorset, UK).

### Tissue array Immunohistochemistry (IHC)

The gastric carcinoma tissue microarray (130 cases/390 cores) contained 83 cases of gastric carcinoma, 27 cases of esophagogastric junction carcinoma and 20 cases of benign tissue samples including adjacent tissue (10cases) and chronic gastritis tissue (10cases) (OD-CT-DgStm01-007, US Biomax, Inc, Rockville, MD, USA). The clinicopathological characteristics including pathological stage, grade, and nodal status were provided on the supplier's website. Standard indirect biotin-avidin immunohistochemical analysis was used to evaluate the EphB2 protein expression. Briefly, prior to staining, slides were placed in an oven set no higher than 55˚C for 1 day to help the sections adhere to the slide. The tissue array section was then dewaxed with xylene and ethanol, and gradually hydrated. Antigen retrieval was performed by placing slides in a plastic container and covered with antigen retrieval buffer, in the microwave on full power for 20 minutes. The rabbit anti-EphB2 antiserum with working concentration (1mg/ml) was incubated at 4˚C overnight. The slides were then washed with Tris Buffered Saline (TBS), and incubated with a universal biotinylated secondary antibody (ABC Elite Kit, Vectastain Universal, PK-6200, Vector Laboratories, CA, USA) for 30 minutes. Following washing with TBS, the sections were incubated with avidin-biotin-peroxidase complex (ABC) for 30 minutes. The 3, 3'-diamino-benzidine (DAB) substrate (5 mg/ml) was used to develop the final reaction product. The sections were then rinsed in water, counterstained with Gill's hematoxylin (Vector Laboratories) and dehydrated through a series of graded alcohols, cleared in xylene and mounted in DPX/Histomount (Merck Millipore, UK). Images were captured using an EVOS FL Auto 2 Cell Imaging System (ThermoFisher Scientific).

All IHC images were manually read by two pathologists who were blinded to the clinical information. The semi-quantitative scoring method was employed to evaluate the intensity of EphB2 positive expression scores (0: negative; 1: weak; 2: moderate; 3: strong). The percentage of the staining was scored. The two scores were added (0: none, 1: 1% to 33%; 2: 34% to 66%; 3: 67% to 100%). The total scores of 0-2 were considered as negative and 3-6 as positive.

### Patients and clinical data

Gastric adenocarcinoma and Siewert type III gastro-oesophageal junction adenocarcinoma tissues (n=171) with matched adjacent background tissues (n=97) were collected immediately after surgical resection at the Beijing Cancer Hospital with informed consent from the patients. The patient selection criteria in this study were defined as follows: 1. Aged from 18 years old; 2. Histologically proven gastric cancer; 3. Subjected to surgical resection without any prior treatment. The tissue samples were stored at -80˚C at the Tissue Bank in Peking University School of Oncology with a record of the relevant clinical and histopathological data. All protocols were reviewed and approved by the Beijing Cancer Hospital Research Ethics Committee (MTA10062009). The clinicopathological data of our GC cohort were shown in Table 1.

### RNA isolation and quantitative real-time PCR (qPCR)

Total RNA was extracted from fresh frozen gastric tissues and cultured GC cell lines using TRI reagent (Sigma-Aldrich, Dorset, UK) following the manufacturer's instruction. First strand cDNA was synthesized from 1 μg RNA using a first-strand DNA synthesis kit (Bio-Rad, Hemel Hempstead, UK). Quantitative analysis of EphB2 mRNA expression in GC tissues was performed using Amplifluor™‑based real-time PCR, in which a 6-carboxy-fluorescine-tagged Uniprimer™ (Biosearch Technologies, Inc.) was used as a probe along with a pair of specific primers with an addition of a Z-sequence (actgaacctgaccgtaca) to the 5'-end of the reverse primer. The quality of cDNA samples was verified using glyceraldehyde-3-phosphate dehydrogenase (GAPDH) as a housekeeping gene. The primer sequences for qPCR were as follows:

EphB2-forward: TTGAGAATGGCACCGTCT; EphB2-reverse: ACTGAACCTGACCGTACAGATGGGACAGTGGGTACAG; GAPDH-forward: AAGGTCATCCATGACAACTT; GAPDH-reverse: ACTGAACCTGACCGTACAAGCCATCCACAGTCTTCTG.

### Kaplan-Meier survival analysis

We performed the survival analysis using the gene expression data obtained from our cohort. As a comparison, the association between EphB2 gene expression and survival of patients with GC was assessed using an online Kaplan-Meier survival analysis tool (http://kmplot.com) 17. This online tool allowed us to analyze the OS from 876 cases, FP from 641 cases and PPS from 499 cases of GC by pooling all the published Affymetrix gene expression microarray data (EphB2 Probe set ID: 211165_x_at, 209588_at, 210651_s_at and 211165_x_at).

### Transfection with EphB2 plasmid

The CRISPR activation plasmid specific for EphB2 was obtained from Santa Cruz Biotechnology, INC. AGS and HGC27 cells were transfected with 1 µg of the CRISPR activation plasmid specific for EphB2 (Santa Cruz Biotechnology Inc., Santa Cruz, CA, USA) in Opti-MEM Medium using Lipofectamine™ 3000 Transfection Reagent (ThermoFisher, USA) according to the manufacturer's instruction.

### Western blot analysis

Protein samples following cell lysis were separated using SDS-PAGE followed by electroblotting onto a nitrocellulose membrane (Millipore Ltd, Watford, UK). The proteins were then probed with the anti-EphB2 antibody (1:1,000, Signalway Antibody SAB, Pearland, USA) and anti-GAPDH antibody (1:5,000, Santa Cruz Biotechnology, Inc.) as a house keeping control, followed by a peroxidase-conjugated secondary antibody (1:2,000, Sigma). Protein bands were visualized using a chemiluminescence detection kit (Luminata, Millipore) and photographed with the gel documentation system G: Box (Syngene Europe, Cambridge, UK).

### Flow cytometric analysis

Cells were harvested and washed in ice cold PBS and resuspended in 1X Annexin-binding buffer at a density of 1x10^6^ cells/ml after centrifugation. FITC Annexin V (5 µl) and PI working solution (1 µl) (100 µg/ml) (Molecular Probes, Eugene, OR, USA) were added to 100µl of the cell suspension. After incubation for 30 minutes at room temperature, 400 µl of 1X Annexin‑binding buffer was added, mixed gently and the samples were kept on ice. The stained cells were immediately analyzed by flow cytometry (FACS Canto^TM^ II, BD BioSciences, San Jose, CA, USA).

### *In-vitro* cell proliferation assay

Cells were seeded into 96-well plates in complete growth culture medium at a density of 5000 cells/well. Following cultivation for 24 and 48 hours, respectively, cells were fixed with 4% formaldehyde followed by staining with 0.5% crystal violet. The crystal violet was then dissolved in 10% acetic acid prior to a colorimetric detection at a wave length of 580 nm using the ELx800 spectrophotometer.

### *In-vitro* invasion assay

The 24-well transwell plate inserts with 8-μm pores (Greiner Bio-one, Frickenhausen, Germany) were pre-coated with 30 μg/well of Matrigel (BD Bioscience, Oxford, UK) and air-dried. Cells at a density of 30,000 cells/well were seeded to each well after 60-min rehydration of the Matrigel using PBS. After incubation for 24 h, cells that invaded through the Matrigel matrix to the other side of the insert were detached with an enzyme-free cell dissociation solution and stained with the DNA-binding fluorescent stain Hoechst 33342 (Sigma). The cell number was then determined by measuring fluorescence signal (excitation, 340-380 nm; emission, >415 nm) using a 96-well plate reader (GloMax, Promega, oxford, UK).

### Cell-matrix adhesion assay

The 96-well culture plates were pre-coated with 5 μg/well of Matrigel and air‑dried. Following the rehydration, 20,000 cells were seeded into each well. After incubation for 40 min, non-adherent cells were washed-off using PBS. The number of adherent cells was counted after fixation and staining using crystal violet as described above.

### Electric cell‑substrate impedance sensing (ECIS) analysis

The Electric Cell-Substrate Impedance Sensing (ECIS™) 9600 system (Applied Biophysics, Troy, NJ) was used to analyze the attachment and migratory behaviour of GC cells. Briefly, AGS and HGC27 cells were seeded onto ECIS 96W1E arrays and the migration of cells to the culture surface between the two electrodes was monitored by measuring electrical resistance. Once a confluent monolayer had been formed, the cells were damaged by applying an electric current (2600 µA, 60 kHz) for 20 seconds to create a break in the cell monolayer. The rate of change in impedance as cells migrated back onto the electrode sensing site was subsequently monitored and analyzed.

### Wound healing assay

Cells were cultivated until they reached confluency. A scrape in the cell monolayer was made in one direction with a fine pipette tip. The wounded cell monolayers were washed with PBS to remove cell debris. The remaining gaps which indicated the wound-healing migration ability of the cells were analyzed using an inverted microscope at different time points.

### Bioinformatic analysis

The gene expression correlation in our GC cohort was analyzed using the Pearson correlation test. The enrichment analysis of the significantly correlated genes was performed using the Cytoscape program 18 and the ConsensusPathDB plugin 19, 20. The protein associated interaction network was predicted using the STRING program 21. To confirm the observation from our patient cohort, we also performed pooled bioinformatic analysis of mRNA expression using the publicly available Cancer Genome Atlas Stomach Adenocarcinoma (TCGA-STAD) data base with 415 GC tumour samples and 35 normal gastric tissue controls.

### Statistical analysis

All statistics were conducted with the R language (Version 3.6.1) 22 using RStudio (Version 1.2.1335. R Studio Inc., Boston, Massachusetts, USA). Kruskal-Wallis or Wilcoxon test was used for the comparison of non-parametric data, while ANOVA or t-test was used if data pass the Shapiro normality test. Results were considered to be statistically significant when p<0.05.

## Results

### Protein levels of EphB2 are higher in human GC tissue

**Figure 1:**
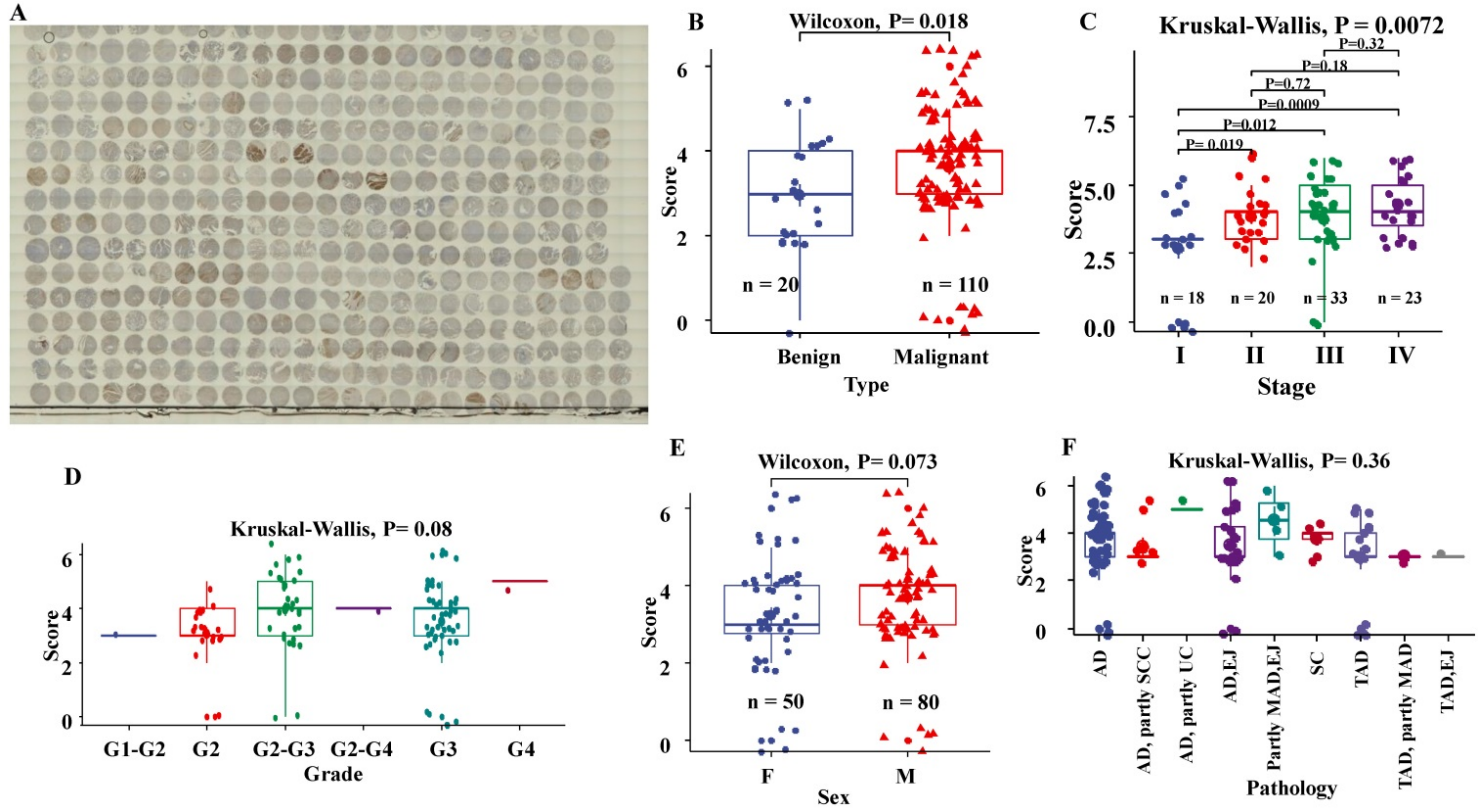
** Expression profile of EphB2 protein in a tissue microarray of GC by immunohistochemical analysis.** (A) Thumbnail image of the tissue microarray after IHC with a DHX36 antibody. (B) Protein levels of EphB2 between benign and malignant tissue types. (C) Protein levels of EphB2 among GC stages. (D) Protein levels of EphB2 among GC grades. (E) Protein levels of EphB2 between sexes. (F) Protein levels of EphB2 among pathological subtypes. AD, adenocarcinoma; SC, Stomach carcinoma; SCC, signet ring cell carcinoma; UC, undifferentiated carcinoma; MAD, mucinous adenocarcinoma; EJ, esophagogastric junction; TAD, Tubular adenocarcinoma.

We assessed the association of the EphB2 staining scores following IHC with the clinicopathological variables of the tissue microarray including tissue type, Pathological diagnosis, pTNM stage and histological grade. As shown in Fig.1, The staining scores of EphB2 were significantly higher in malignant tumour tissues than benign ones (P= 0.018). Also, the protein levels of EphB2 in late stages (II, III and IV) tended to be upregulated (P= 0.0072). There was no significant association of the EpHB2 IHC staining levels with the grade (P= 0.08), sex (P= 0.073) and pathological types (P= 0.36) of GC.

### Higher expression of EphB2 in transcript levels of human GC

Transcript levels of EphB2 were determined in our GC cohort using real-time qPCR. The results showed that EphB2 expression was significantly upregulated in gastric tumours compared to normal tissue (P=0.0020) (Table 1). The differentiated expression level EphB2 in GC was also observed between genders, male GC patients exhibited higher expression than female (P=0.0056). The transcript level of EphB2 in the stages III+IV stages was significantly higher than the stages of I+II (p=0.0480), which appeared to be in line with the observation of the protein expression by IHC. There was also a higher gene expression of EphB2 in tissues from patients who received radical surgical treatment than those who received Non-Radical surgery. The higher mRNA expression of EPHB2 in gastric tumours was also confirmed by the data analysis of the TCGA-STAD database as shown in Supplement Figure 3(p<0.0001).

### Association of EphB2 expression with the survival of patients with GC

**Figure 2:**
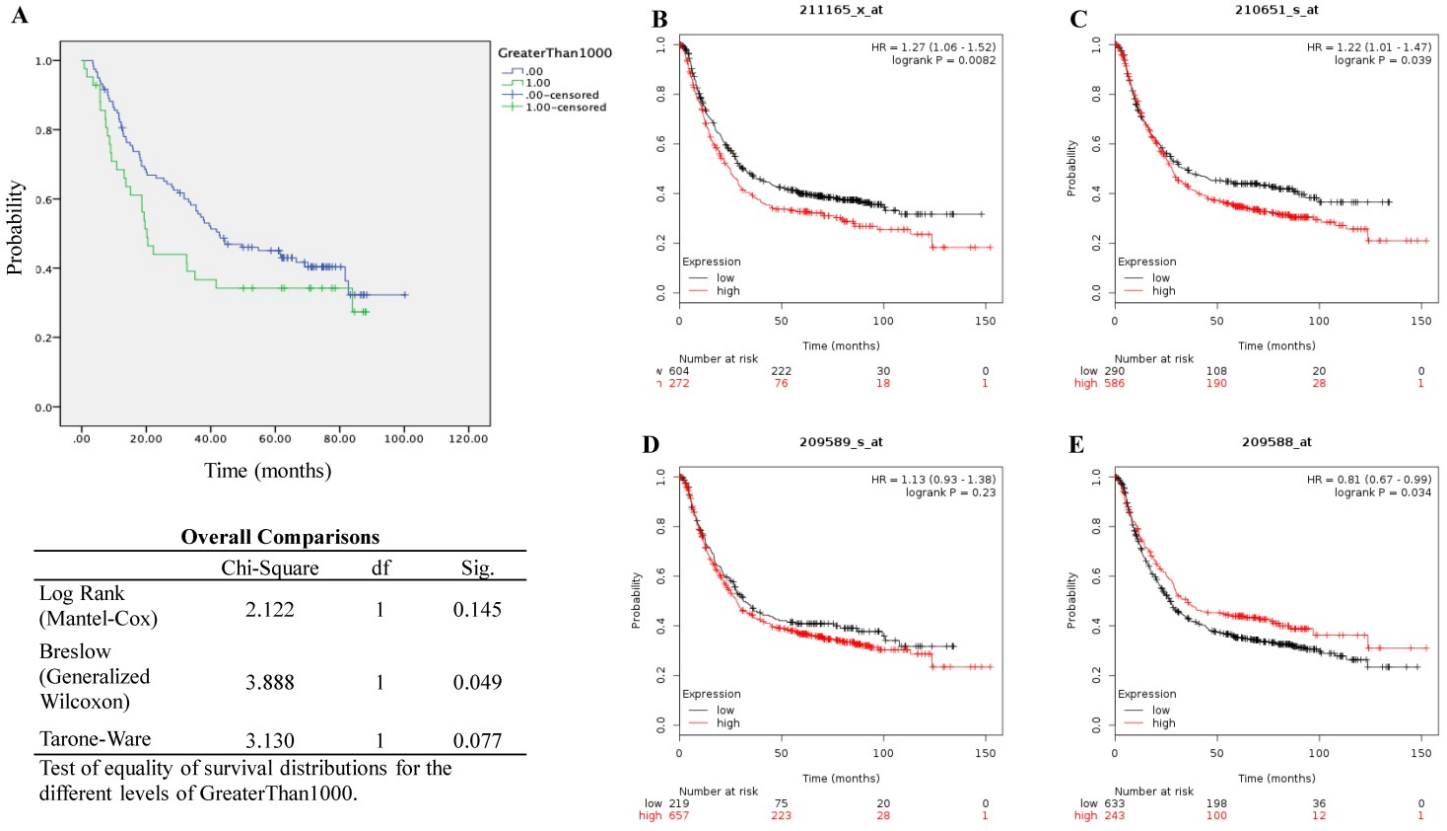
** Survival analysis of patients with GC based on the mRNA levels of EphB2.** (A) Overall survival analysis using the qPCR data from our GC cohort. (B-E) Kaplan-Meier survival analysis (KMplot, http://kmplot.com) by pooling 876 cases that were subjected to expression profiling using Affymetrix gene expression microarray (EphB2 Probe set ID: 211165_x_at, 209588_at, 210651_s_at and 211165_x_at). Auto-selected cutoff values of EphB2 gene expression were applied in the analysis.

We performed the Kaplan-Meier survival analysis using the gene profile data from our cohort. It showed that high transcript expression of EphB2 appeared to be linked with poor overall survival (OS) although the statistical significance was not reached according to the log Rank test (P=0.145) despite the P value by Breslow test is 0.049 (Figure 2A). As a comparison, we also evaluated survival using the online KMplot database (Figure 2B-2E). Within the four probe set IDs of EphB2, two of them indicated that high gene expression of EphB2 is associated with poor OS including 210651_s_at (P=0.0082) and 211165_x_at (P=0.039), one of them indicated opposite association (209588_at, P=0.034), while one of them did not show any significance (209589_s_at, P=0.23).

### CRISPR-mediated activation of EphB2 in GC cells

**Figure 3:**
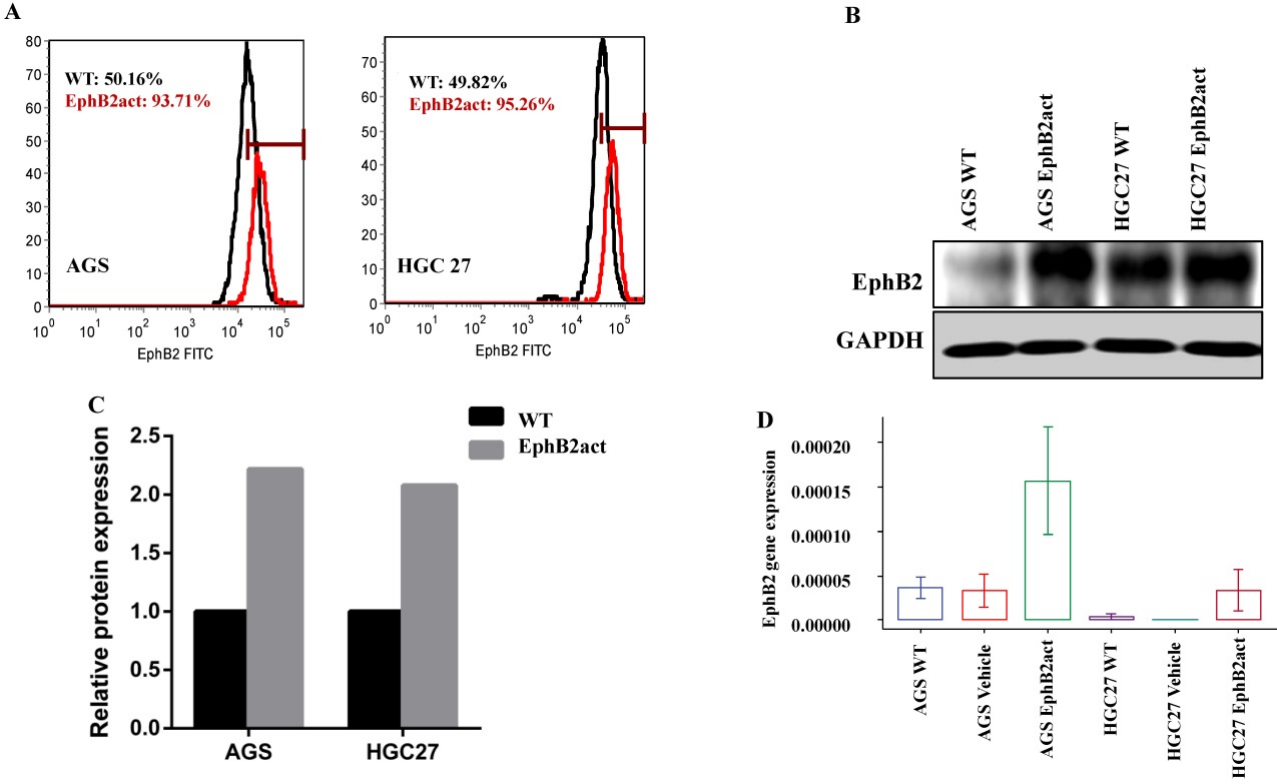
** Validation of EphB2 expression after CRISPR-mediated activation in AGS and HGC27 cell lines.** (A) Flow cytometric analysis of the protein levels of EphB2 in cells. (B and C) Western bolt analysis of the protein levels of EphB2 in cells. (D) mRNA levels of EphB2 in GC cells indicated by qPCR.

As the endogenous expression levels of EphB2 in HGC27 and AGS wild type cells were relatively low after initial evaluation (Supplement Figure 2), we attempted to activate the EphB2 expression using the CRISPR-mediated EphB2 activator. The change of the EphB2 expression was examined using qPCR, Flow cytometry and Western blotting in comparison with the WT control. The Flow cytometric data indicated that there was a dramatic increase in EphB2 expression in cells after treatment with the EphB2 activator in both AGS and HGC27 cells (Figure 3A). The Western blotting confirmed the result of flow cytometry (Figure 3B and 3C). The qPCR data showed that there was a higher expression of EphB2 gene in the EphB2 activation group compared with the controls (4- to 7-fold) (Figure 3D).

### Activation of EphB2 enhances the migration ability of the GC cells

**Figure 4:**
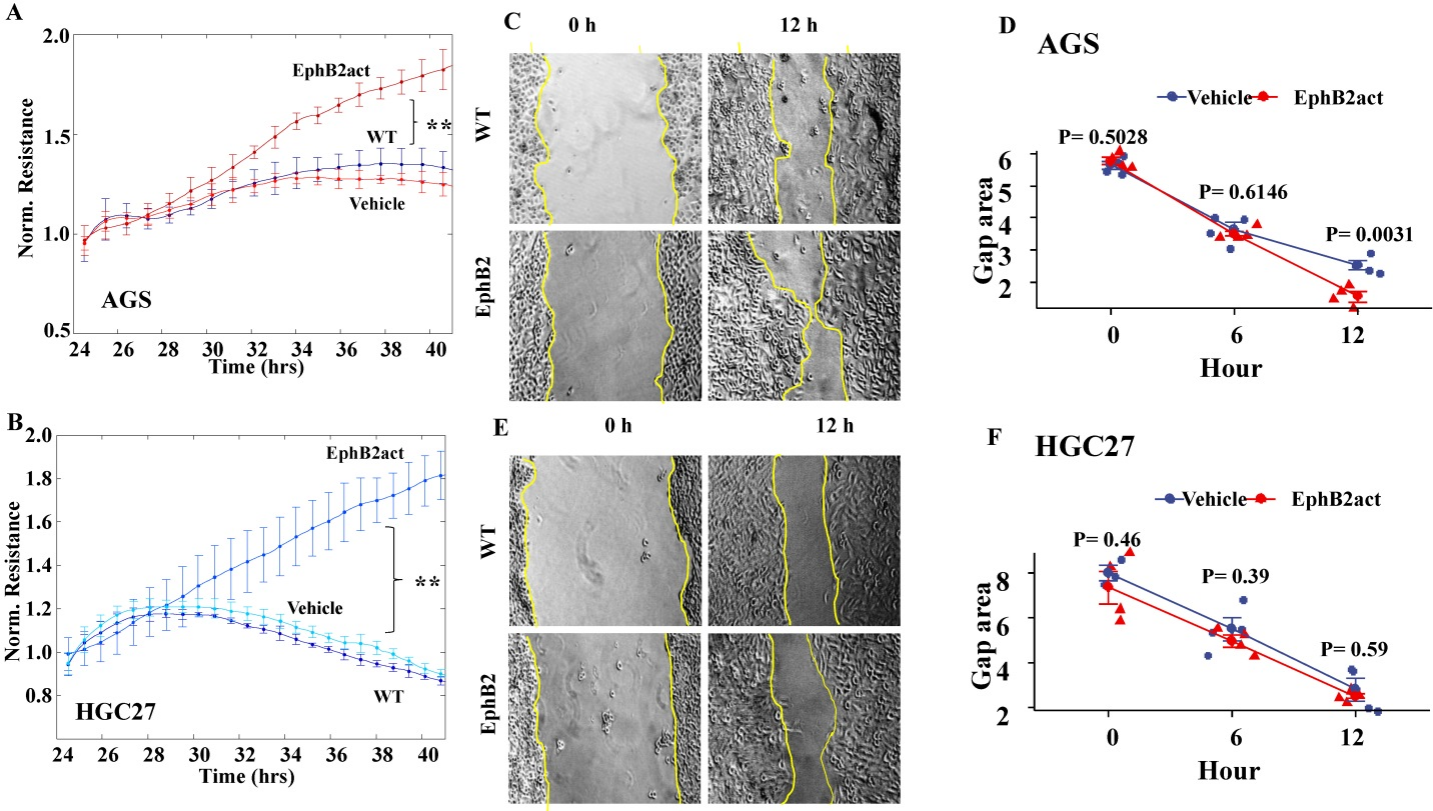
** Effect of EphB2 activation on the migration of GC cells.** (A and B) Migration of the GC cell lines indicated by ECIS in AGS and HGC27. * P<0.05 and ** P<0.01 by repeated measures ANOVA. (C) Representative images of the wound-healing migration ability of AGS cells. (D) Migration ability of AGS cells indicated by the closure of the gap area after scratch assay. (E) Representative images of the wound-healing migration ability of HGC27 cells. The cell monolayer gaps were highlighted using yellow lines. (F) Migration ability of HGC27 cells indicated by the closure of the gap area after scratch assay.

As indicated by the ECIS system (Figure 4A and 4B), the migration levels of both gastric cells were enhanced significantly in cells with EphB2 activation compared to the vehicle and WT controls (p<0.01, respectively). The scratch wound-healing assay was performed to evaluate migration in a directly visible but low-accuracy manner. As shown in Figure 4C and 4D, EphB2 activation for 12 hours accelerated the closure of the gap area indicating the elevated migration ability of AGS (P=0.0031 vs WT). However, there was no difference in terms of the wound healing closure speed in HGC27 cells (P=0.59) (Figure 4E and 4F).

**Figure 5:**
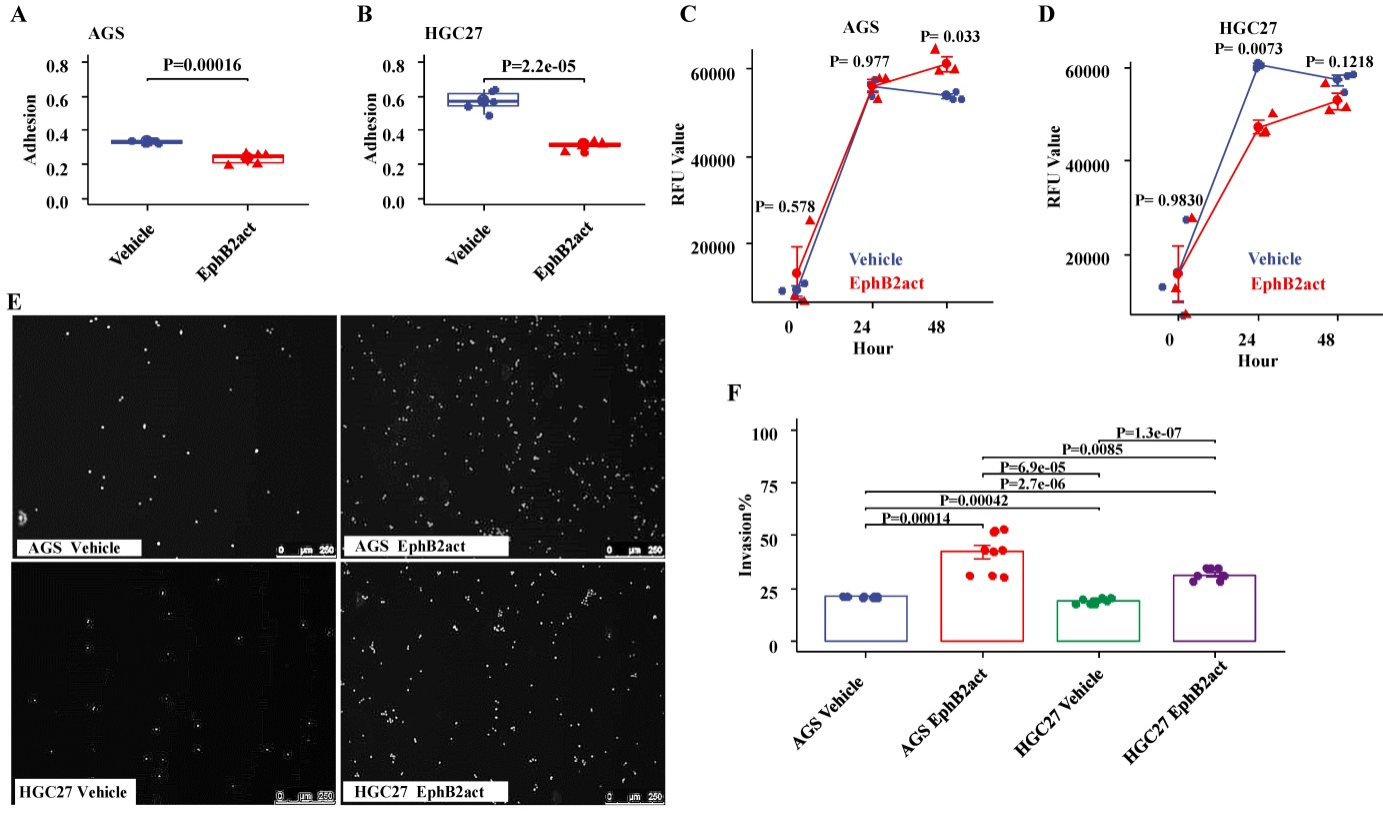
** Effect of EphB2 activation on adhesion, proliferation and invasion of GC cells.** (A and B) Adhesion ability of GC cells indicated by crystal violet staining. (C and D) Proliferation levels of GC cells indicated by the Alamar Blue Assay. (E) Representative images of cell invasion indicated by nuclear staining with Hoechst 33342. (F) Comparison of cell invasion by fluorescence plate reading and normalization using the number of seeding cells.

Activation of EphB2 reduces the adhesion ability of the GC cells. The effect of EphB2 activation on the adhesive ability of GC cell lines was evaluated using an *in vitro* matrix adhesion assay. As shown in Figure 5A and 5B, EphB2 activation reduced the adhesion ability of both AGS and HGC27 cells significantly (p<0.001 vs. the Vehicle control, respectively). The effect of EphB2 activation on cellular proliferation was also examined. It appeared that EphB2 activation increased the proliferation of AGS cells after 48 hours (P= 0.033), but decreased the proliferation of HGC27 cells after 24 hours (P= 0.0073) compared to the vehicle control (P=0.013 and P=0.000), and no time dependence change was observed (Figure 5C and 5D).

### Activation of EphB2 promoted the invasion of the GC cells

As shown in Figure 5E and 5F by an invasion assay, EphB2 activation led to an accelerated level of invasion ability of both AGS cells (P=0.00014 vs vehicle) and HGC27 cells (P<0.0001 vs vehicle). Further, it appeared that the increase of invasion in AGS cells was higher than HGC27 cells in response to EphB2 activation (P=0.0085).

### Transcriptional regulatory network of EphB2 in GC

**Figure 6:**
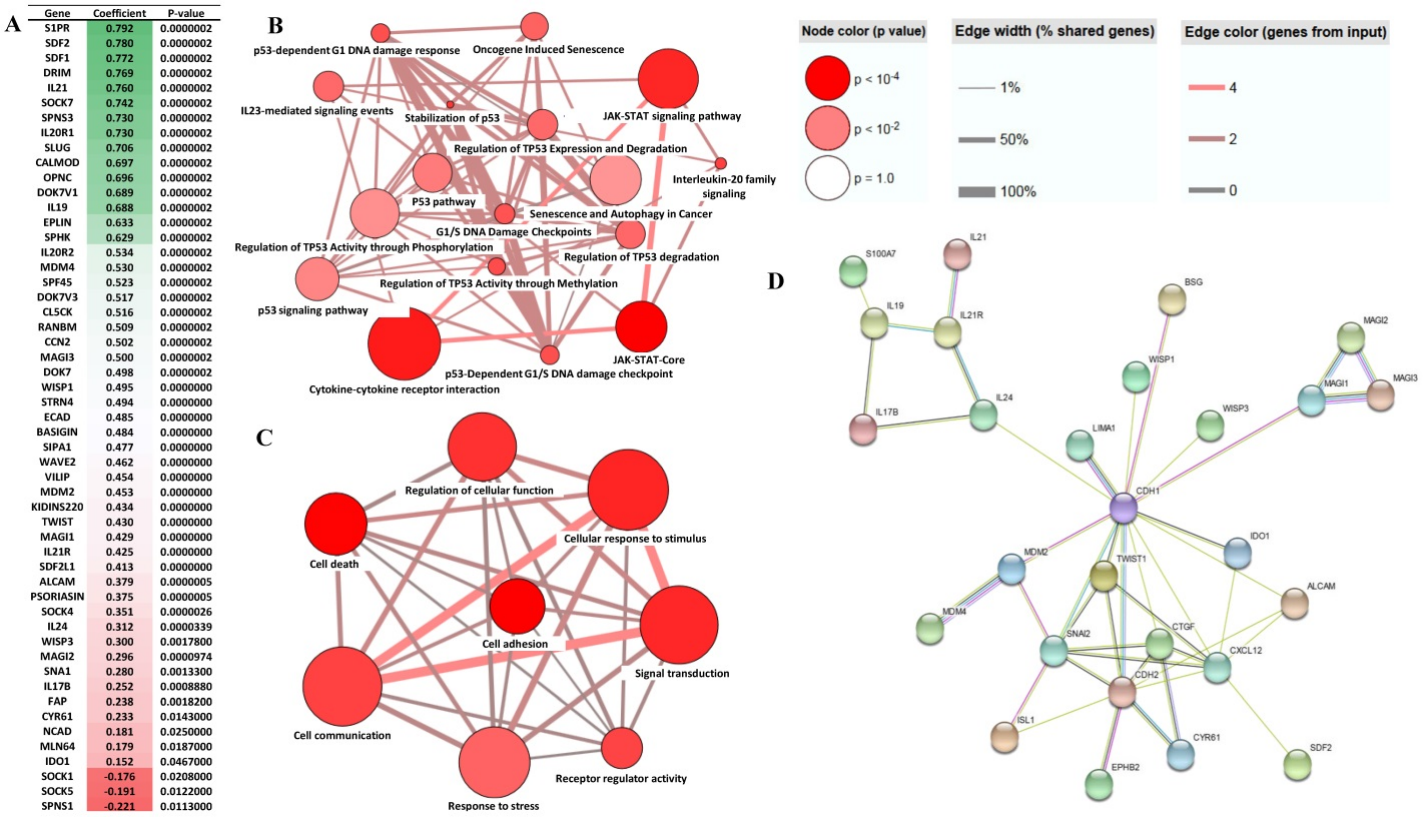
** Analysis of EphB2 correlated genes in GC.** (A) Genes which correlate with EphB2 in the GC cohort (p<0.05 by Pearson test). (B-D) Significant signaling pathways and cellular functions of the EphB2-correlated genes in GC which are determined using the ConsensusPathDB-human database system (http://cpdb.molgen.mpg.de/CPDB). (E) The interaction network of the EphB2-correlated genes in GC identified using the STRING web server (http://string-db.org).

To establish the Transcriptional regulatory network EphB2 is involved in GC, we preformed the gene correlation analysis using the gene profiling data from our cohort. This enabled us to identify a panel of the differential genes which correlated with EphB2 either positively or negatively (p<0.05) (Figure 6A). To determine the biological pathways and possible cellular functions EphB2 is involved in through interacting with the correlated genes in GC, we performed a gene ontology (GO) biological process enrichment analysis. As shown in Figure 6B, EphB2 is mainly involved in cytokine-cytokine receptor interaction, p53 regulation and signaling pathway and JAK-STAT signaling pathway. And the affected cellular functions include signal transduction, cell communication, response to stimulus and stress, cell death and cell adhesion (Figure 6C). The protein interaction network determined by STRING identified that EphB2 may interact with the CDH2 signaling linage initially. And CDH2 is associated with a panel of GC biomarker proteins such as CDH1, SNAI2, CTGF and TWIST1 (Figure 6D).

## Discussion

In this study, we evaluated the EphB2 expression in GC tissues using tissue microarray and a cohort we obtained. Higher levels of EphB2 expression are observed in GC tumour tissue than in adjacent normal or benign gastric tissue specimens, which include the transcript level and protein expression. And the transcript level of EphB2 in T3+T4 was significantly higher than the group of T1+T2. We therefore demonstrate that EphB2 is overexpressed in GC. Also, our survival analysis suggests that high gene expression of EphB2 may be associated with poor prognosis of patients with GC. The clinical significance of EphB2 has been investigated in some other solid tumour types. The clinical studies in breast and lung cancers seem in line with our observation in GC. In breast cancer, 82% (77/94) patients show moderate and strong EphB2 protein expression, and the increased level of EphB2 expression correlated with poor overall survival of patients 21. And in lung adenocarcinoma, high expression of junctional adhesion molecule-A (JAM-A) and EphB2 can together predict the poor overall survival and high mortality rate of patients, suggesting its prognostic value 23. However, EphB2 may play a diverse role in colorectal cancer. A study on tissue microarray shows that low EphB2 expression is linked with more advanced tumour stages, poor differentiation, poor overall survival and disease-free survival of patients with colorectal cancer, which may contrast with EphB4 9.

Additionally, we found a differentiated gene expression of EphB2 in GC between sexes, tissue samples from the male exhibited higher expression levels than female samples. EphB2 expression appears to be temporally and spatially regulated in the developing mouse genital tubercle (GT) and differs between sexes, male pattern of expression can be induced in the female GT by dihydrotestosterone exposure. Given dihydrotestosterone exposure alters EphB2 expression, this indicates that EphB2 is a candidate androgen regulated gene 24.

We show in this study that EphB2 activation significantly decreases the adhesion ability of GC cells. By performing correlation gene analysis, we identified several genes which are involved in cell adhesion such as ALCAM 25, SIPA1 26, WISP1 and WISP3. Meanwhile, EphB2 activation accelerates both the migration and invasion of gastric cancer cells. In other cancer cell lines, there is evidence that EphB2 regulate migration by modulating the EMT process 27. This may also be the case because there is a positive correlation between EphB2 and EMT markers including SNAI1 and ECAD. Also GC cell invasion is promoted by the activation of EphB2. It is known that EphB2 has a pro-invasion role in other types of cancer cells 7, 28. It is known that AGS is a poorly differentiated gastric adenocarcinoma cell line, whereas HGC27 is an undifferentiated GC cell line. Also AGS is a p53 wild-type GC cell line, while HGC27 is a p53 mutant cell line 29. P53 activation transcriptionally regulates the expression of its target genes to modulate various cellular processes in response to various tumour-microenvironment stress signals, including apoptosis and cell cycle arrest 30. However, It appears that the role of EphB2 is GC cells is less likely p53 dependent. Collectively, as EpB2 play a role in decreasing adhesion and accelerate migration and invasion of GC cells, it is likely that EphB2 may promote metastasis of GC. This can be indirectly supported by studies on some other cancer types such as cholangiocarcinoma 31, cutaneous squamous cell carcinoma 32 and cervical cancer 27.

## Conclusion

Our research shows that the levels of EphB2 are significantly elevated in tumour tissue including gene and protein expression. And the high level of EphB2 expression correlates with poor OS. Therefore EphB2 represents as a novel independent prognostic marker in patients with GC. The activation of EphB2 in GC cells enhanced the malignant properties of GC cells by reducing adhesion but accelerating the migration and invasion abilities. The results therefore indicate that EphB2 plays a pro-tumour role in GC and present the therapeutic potential for the treatment of GC.

## Supplementary Material

Supplementary figures.

## Figures and Tables

**Table 1:** Gene expression of EphB2 in the gastric cancer cohort.

Variable	N	Mean	Median	STD	SE	*p-*value
Tumour	171	31785	397	93022	7114	
Normal	97	8267	258	24226	2460	**0.0020**
Male	129	38643	961	105509	9290	
Female	42	10718	149	22859	3527	**0.0056**
Gastric	132	25419	194	84019	7313	
Cardiac	32	53728	8183	119996	21212	0.2100
Intestine	2	207	207	269	190	**0.0008**
T1	8	21909	773	45418	16058	
T2	10	5319	107	13270	4196	0.3500
T3	28	32967	1671	128974	24374	0.7100
T4	120	35470	356	91321	8336	0.4700
T1+T2	18	12692	309	31852	7508	
T3+T4	148	34996	742	99032	8140	**0.0480**
N0	34	35044	491	121607	20855	
N1	22	22270	27	86539	18450	0.6500
N2	40	36598	249	101016	15972	0.9500
N3	72	31419	1218	77142	9091	0.8700
N1+2+3	134	31463	359	85869	7418	0.8700
TNM1	13	14319	584	36173	10032	
TNM2	26	39416	268	137294	26926	0.3900
TNM3	122	33653	558	89396	8094	0.1400
TNM4	4	22555	638	44262	22131	0.7500
TNM1+2	39	31050	372	113833	18228	
TNM3+4	126	33301	558	88243	7861	0.9100
Diff-HM	5	58252	6449	121072	54145	
Diff-M	30	48140	481	137422	25090	0.8700
Diff-ML	40	28756	3446	48435	7658	0.6200
Diff-L	72	26609	194	95919	11304	0.6000
Embo-No	76	35744	198	111641	12806	
Embo-Yes	86	30058	1104	78624	8478	0.7100
Non-Radical	40	14573	67	38708	6120	
Radical	130	37317	946	104021	9123	**0.0400**
Alive	68	39752	221	116638	14144	
Died	102	26775	948	73909	7318	0.4200
Disease-Free	61	40243	224	121097	15505	
Metastasis	7	35473	117	72445	27382	0.8800
Died of GC	102	26775	948	73909	7318	0.4300
