# Supplementary material for: EphB2 represents an independent prognostic marker in patients with gastric cancer and promotes tumour cell aggressiveness
Source: J Cancer. 2020 Feb 21;11(10):2778–87. doi: 10.7150/jca.38098 (PMC7086246; doi:10.7150/jca.38098)
Supplement: Supplementary file 1 — Supplementary figures. [file jcav11p2778s1.pdf]

Supplement Figure 1. Representative images showing the different scores of IHC staining using a DHX36 antibody. (A) Staining Intensity. (B) Positive percentage.

Supplement Figure 2. mRNA expression of EPHB2 in cancer cell lines. (A) mRNA expression profile of EPHB2 in cancer cell lines on the website of https://www.proteinatlas.org. (B) mRNA expression of EPHB2 in the gastric cancer cell lines in our lab by qPCR assay using HUVEC cells as external positive control.

Supplement Figure 3. Pooled data analysis of mRNA expression using the publicly available Cancer Genome Atlas Stomach Adenocarcinoma (TCGA-STAD) data base. The database constains 415 GC tumour samples and 35 normal gastric tissue controls. (A) mRNA expression of EPHB2 between GC tumou samples and normal tissue controls. (B) Comparison among GC stages. (C) Comparison among GC higtological types. (D) Comparison among GC grades. (D) Comparison between the female and the male samples. The Kruskal-Wallis test was used to evaluate the difference among multiple groups, while the Wilcoxon test was used to compare two groups. The difference was considered statistically significant when $p<0.05$.

A

Staining Intensity

Score 0 **Score 1** **Score 2** **Score 3**

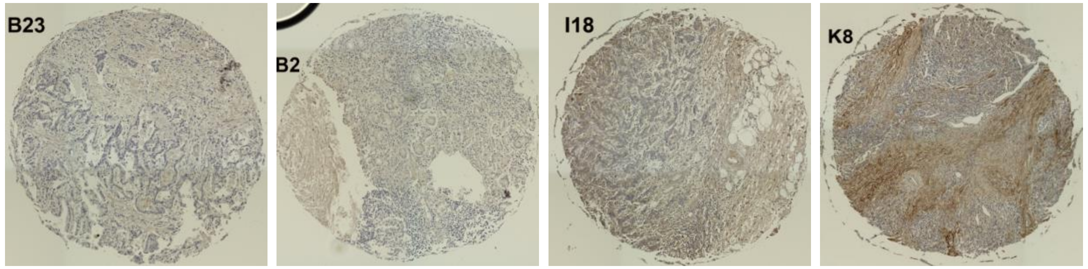

B

Positive percentage

Score 0 **Score 1** **Score 2** **Score 3**

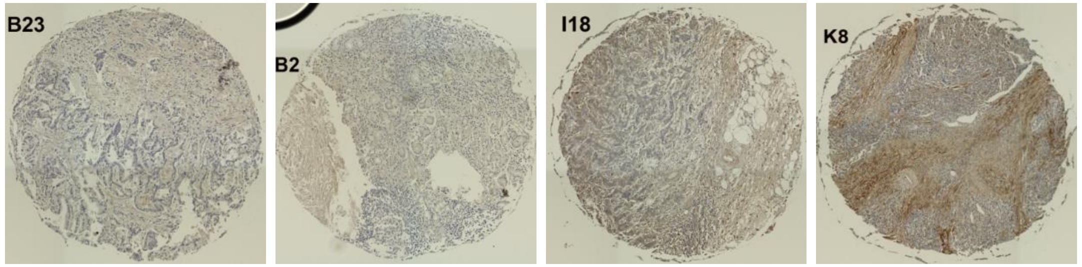

Supplement Fig. 1

A

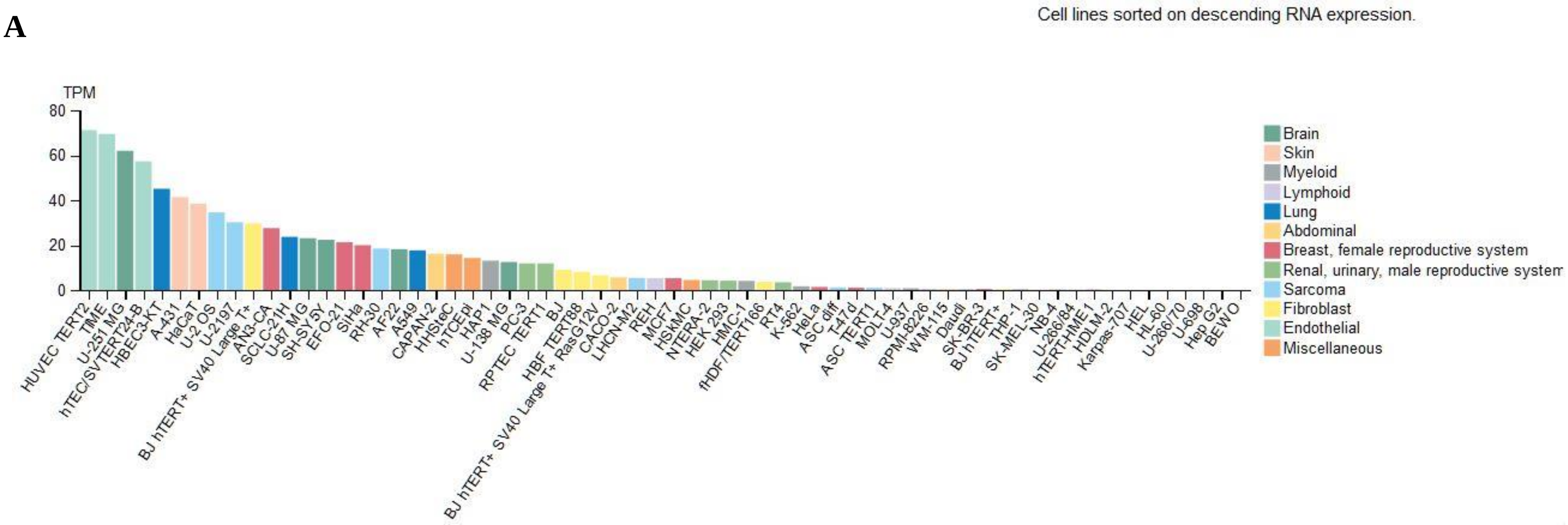

B

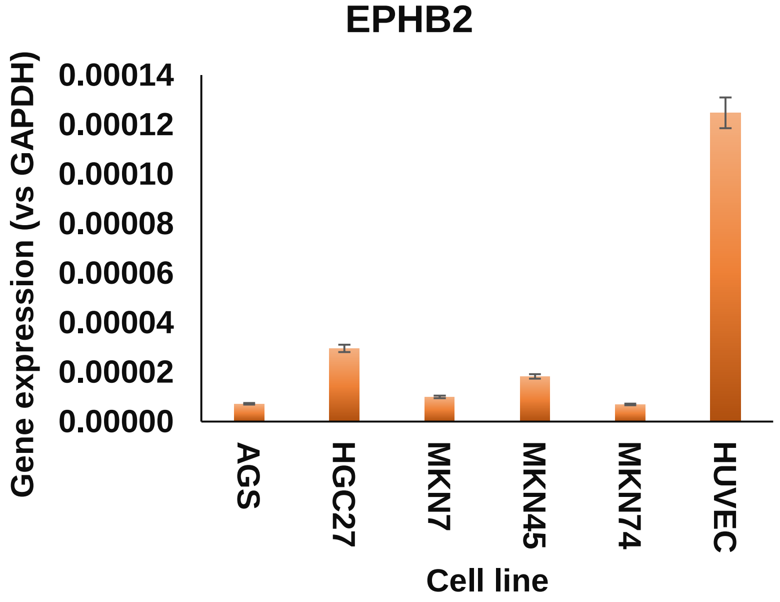

Supplement Fig. 2

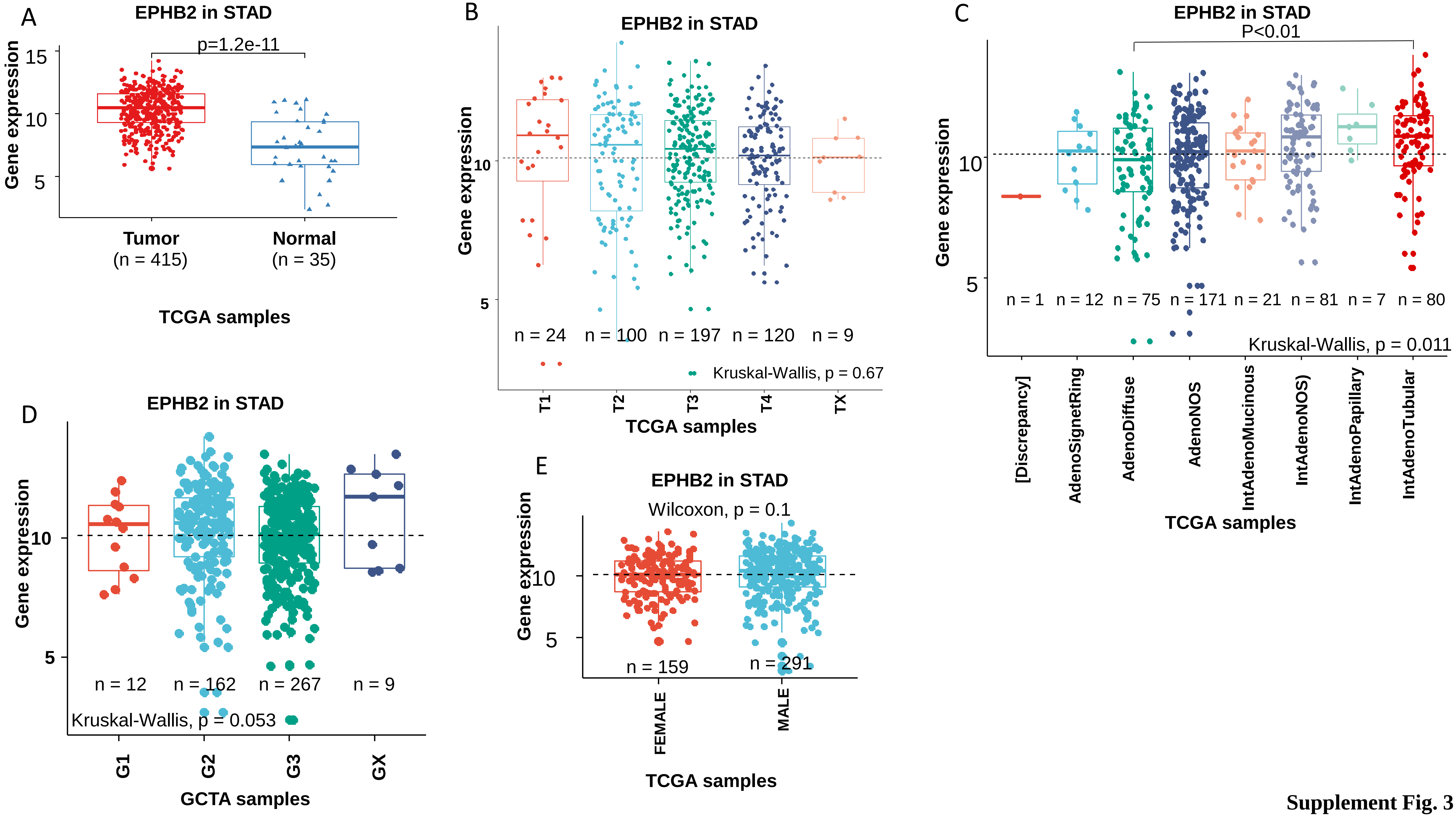

Supplement Fig. 3
